# Genetic Damage Induced by Accidental Environmental Pollutants

**DOI:** 10.1100/tsw.2006.206

**Published:** 2006-09-25

**Authors:** Beatriz Pérez-Cadahía, Blanca Laffon, Eduardo Pásaro, Josefina Méndez

**Affiliations:** ^1^ Toxicology Unit, University of A Coruña, Edificio de Servicios Centrales de Investigación, Campus Elviña s/n, 15071-A Coruña, Spain; ^2^ Department of Cell and Molecular Biology, University of A Coruña, Faculty of Sciences, Corampus A Zapateira s/n, 15071-A Coruña, Spain

**Keywords:** accidental pollutants, genotoxicity, micronucleus (MN) test, sister chromatid exchanges (SCE), comet assay

## Abstract

Petroleum is one of the main energy sources worldwide. Its transport is performed by big tankers following some established marine routes. In the last 50 years a total amount of 37 oil tankers have given rise to great spills in different parts of the world, *Prestige* being the last one. After the accident, a big human mobilisation took place in order to clean beaches, rocks and fauna, trying to reduce the environmental consequences of this serious catastrophe. These people were exposed to the complex mixture of compounds contained in the oil. This study aimed at determine the level of environmental exposure to volatile organic compounds (VOC), and the possible damage induced on the population involved in the different cleaning tasks by applying the genotoxicity tests sister chromatid exchanges (SCE), micronucleus (MN) test, and comet assay. Four groups of individuals were included: volunteers (V), hired manual workers (MW), hired high-pressure cleaner workers (HPW) and controls. The higher VOC levels were associated with V environment, followed by MW and lastly by HPW, probably due to the use of high-pressure cleaners. Oil exposure during the cleaning tasks has caused an increase in the genotoxic damage in individuals, the comet assay being the most sensitive biomarker to detect it. Sex, age and tobacco consumption have shown to influence the level of genetic damage, while the effect of using protective devices was less noticeable than expected, perhaps because the kind used was not the most adequate.

### INTRODUCTION

Humanity has made a lot of progress in the last decades, but parallel to these great advances, serious environmental problems have resulted. Among them, emissions are becoming more and more important, coming from industry in different ways: fumes to the atmosphere, dumps of a great variety of compounds, and several kinds of spills to the rivers and the sea. In this last section, it is important to point out oil spills because of their magnitude and environmental effects. Petroleum is one of the main energy sources worldwide, which entails the need for carrying it from the platforms to ports around the world. Transport is performed by big tankers and follows established marine routes. This fact, added to the bad state of some oil tankers, causes a high number of spills to take place all over the world. In the last 50 years, a total amount of 37 oil tankers have given rise to great spills in different parts of the world, *Prestige* being the last one.

This tanker contained over 77,000 tons of crude oil and wrecked in November 2002 at 130 nautical miles from the coast of Galicia, in the Northwest of Spain. *Prestige* contained heavy oil, resulting from the refining of crude oil that was classified as oil number 6 by the U.S. Environmental Protection Agency (USEPA). It was composed mainly of saturated hydrocarbons (22%), aromatic hydrocarbons (50%), and resins and asphaltens (28%)[1]. Attending to the existing considerations on these kinds of substances, two groups of compounds must be highlighted as especially dangerous agents: volatile organic compounds (VOC) and polycyclic aromatic hydrocarbons (PAH). The first group has been classified as toxic and some of its components are even considered as mutagenic and/or carcinogenic (benzene, styrene, etc.), being their toxicity is aggravated by their high volatility, which facilitates their penetration inside organisms. As for PAH, their main characteristics are also mutagenic and carcinogenic, directing their affinity to the nucleophilic centres of big macromolecules as DNA, RNA, or proteins.

After the *Prestige* accident, a large human mobilisation took place in order to clean beaches, rocks, and fauna, trying to reduce the environmental consequences of this serious catastrophe. For this reason, besides the ecological repercussion, an important human population composed of zone inhabitants, volunteers who came from different points of Spain and even Europe, and hired workers, was exposed to these toxic agents during different periods of time and in different ways. Knowing that genetic material could be one of the main targets of this exposure, it seemed necessary to perform a genotoxicity study to identify and define the repercussion of the exposure on human health. Among the different genotoxicity tests, three were selected in order to better characterize the kind of damage caused: sister chromatid exchanges (SCE), micronucleus (MN) test, and comet assay.

SCE are cytogenetic manifestations of DNA breaks and their cross join in the homologous place in the sister chromatid of a chromosome[2]. It has been suggested that they constitute the display of repair processes of DNA double strand breaks by the homologous recombination path[3]. SCE represent an effect biomarker extensively used from the beginning of Genetic Toxicology due to the fact that they may be induced by numerous genotoxic agents.

Micronuclei are defined as small chromatinic bodies that appear in the cytoplasm by condensation of acentric chromosome fragments or whole chromosomes, lagging behind the cell division[4]. One of the main advantages of the MN technique lies in its ability to detect not only clastogenic damage, but also aneugenic processes. This cytogenetic biomarker has been suggested to provide a reasonable epidemiological evaluation of cancer predictivity[5].

Lastly, comet assay is nowadays one of the more frequently employed genotoxicity tests. This technique is characterized for being a rapid, simple, and sensitive method to detect DNA strand breaks in individual cells. Its alkaline version allows the detection of both single and double strand breaks generated from genotoxic agents and incomplete excision repair processes. Thus, the comet assay represents an adequate complement to SCE and MN cytogenetic tests, since it reflects a more recent type of damage that could be repaired.

In summary, knowing the magnitude of the *Prestige* oil spill, the toxic properties of this complex mixture of compounds, and the great amount of people exposed to it, we have performed a study aimed at determining the level of environmental exposure by means of passive dosimeters, and the possible genotoxic damage induced on the population involved in the different cleaning tasks.

## MATERIAL AND METHODS

### Study Subjects

The study population consisted of 110 individuals classified according to their exposure characteristics in controls (n = 42), volunteers that collaborated on cleaning beaches for five consecutive days for 4 h/day (V, n = 25), hired manual workers who also cleaned beaches during 4 months for 6.5 h/day (MW, n = 20), and hired high-pressure cleaner workers that cleaned oil-contaminated rocks during 3 months for 6.5 h/day (HPW, n = 23). The control population was not exposed to any toxic agent for 4 months prior to the study. Data on lifestyle and consumption habits were registered in a detailed questionnaire.

### Environmental VOC Determination

Environmental VOC determination was performed by means of passive diffusion, employing Perkin Elmer tubes filled with 200 mg of Tenax TA and Radiello® tubes. Dosimeters were distributed among the exposed individuals (28 to V, 19 to MW, and 19 to HPW) who bore them during the whole working shift. Tubes were thermally desorbed and analyses were carried out by gas chromatography coupled to ion trap mass spectrometry (ITMS)[6].

The VOC determined were benzene (B), tetrachloroethene (TCE), toluene (T), *n*-heptane (H), *n*-octane (O), ethylbenzene (EB), *m*+*p*-xylene (m+p-X), *o*-xylene (o-X), styrene (S), isopropylbenzene (IPB), *a*-pinene (a-P), *n*-propylbenzene (PB), *b*-pinene (b-P), 1,3,5-trimethylbenzene (1,3,5-TB), *n*-decane (D), pyrene (P), 1,2,4-trimethylbenzene (1,2,4-TB), and 1,2,3-trimethylbenzene (1,2,3-TB). The sum of the quantities of benzene, toluene, ethylbenzene, *m*+*p*-xylene, and *o*-xylene was considered as the index BTEX, and the sum of the total quantity of all evaluated compounds was considered as the index total VOC (TVOC).

### Blood Sample Collection

Peripheral blood samples were collected in heparinised tubes before the beginning of the working shift, between March and May 2003. Samples were immediately transported to the laboratory in a refrigerated container and stored at 4ºC until their utilisation. Every sample was codified in order to ensure a blind study.

### SCE

Duplicate lymphocyte cultures were set up by adding 0.5 ml of whole blood to 4.5 ml of culture medium (the same used for the MN test). Cultures were incubated at 37ºC for 68 h, and after 24 h of culture initiation, 5-bromo-2’-deoxyuridine was added at a final concentration of 7 μg/ml. Colchicine (2 μg/ml) was added 2 h prior to harvesting to arrest the cells at metaphase. Cells were collected by centrifugation, resuspended in a warm hypotonic solution (0.075 *M* KCl at 37ºC) for 10 min, and fixed in Carnoy (methanol-acetic acid 3:1 v/v). Air-dried slides were prepared and stained with fluorescence plus Giemsa technique, following Perry and Wolff[7]. A total of 50 well-spread, second division metaphases were examined for each experimental concentration and donor, half from each duplicate culture, on a Nikon HFX-DX light microscope by the same scorer, to determine the number of SCE/cell.

### Cytokinesis-Block MN Test

Heparinised whole blood (0.5 ml) was cultured in duplicate at 37ºC in 4.5 ml RPMI 1640 medium supplemented with 15% foetal calf serum, 1% phytohaemagglutinin, 1% L-glutamine (200 m*M*), and 1% penicillin (5000 U/ml)/streptomycin (5000 μg/ml) (all from Invitrogen, Barcelona, Spain). Cultures were maintained for 44 h and then 10 µl of cytochalasin-B were added at a final concentration of 6 µg/ml. Cells were harvested by centrifugation after 72-h culture and submitted to a mild hypotonic treatment with 0.075 m*M* KCl at 4ºC. Then cells were fixed in Carnoy (methanol-acetic acid 3:1 v/v), placed on dry slides, and stained with 4,6-diamidino-2-phenylindole (DAPI). Binucleated cytokinesis-blocked cells and MN were identified on a Leica DM-RXA microscope equipped with a 100 W mercury lamp, according to the criteria of Fenech et al.[8]. Two genotoxic parameters were evaluated by scoring 1000 binucleated cells (500 from each culture): frequency of MN and frequency of binucleated micronucleated cells (BNMN).

### Comet Assay

Mononuclear leukocytes were isolated using BD Vacutainer™ CPT™ Cell Preparation Tubes with sodium heparin (Becton Dickinson), following manufacturer's instructions and stored at –80ºC in freezing medium (50% foetal calf serum, 40% RPMI 1640, 10% DMSO) in a Nalgene® Cryo 1ºC Freezing Container (Nalgene Nunc International) until the time of analysis. Just before performing comet assay, cells were thawed at 37ºC, and viability was evaluated by trypan blue exclusion technique, being higher than 85% in all cases.

The alkaline comet assay was performed basically as described by Singh et al.[9], with minor modifications[10]. Briefly, cells were embedded in 80 µl of 0.5% low-melting-point agarose (LMA) (Gibco BRL, Paisley, Scotland), dropped on agarose precoated microscope slides (two for each donor), and spread using a coverslip. Slides were placed on ice for 10 min, a third layer of 80 µl LMA was applied, and then they were immersed in cold lysing solution (2.5 *M* NaCl, 100 m*M* $Na_2EDTA$, 250 m*M* NaOH, 10 m*M* Tris-HCl, pH 10, with 1% Triton X-100 added just before use) for 1 h at 4ºC. After the lysis slides were left for 20 min in the dark on a horizontal electrophoresis tank placed into an ice bath and filled with fresh electrophoresis solution (1 m*M* $Na_2EDTA$, 300 m*M* NaOH, pH > 13) to cover the slides. Electrophoresis was conducted at 0.83 V/cm for 20 min. Slides were neutralized (0.4 *M* Tris-HCl, pH 7.5) and stained with 60 µl of 5 µg/ml DAPI in antifade solution.

One hundred cells (50 per replicate slide) were examined from each sample using the QWIN Comet software (Leica Imaging Systems, Cambridge, U.K.). Tail length (TL) was evaluated as estimator parameter of DNA damage.

### Statistical Analyses

To check for the existence of significant differences among exposure groups, and to examine the contribution of lifestyle factors to the genotoxicity variables evaluated, analysis of variance (ANOVA) and Tuckey's tests were performed. All statistical analyses were conducted using the SPSS for Windows statistical package, version 14.0 (Chicago, IL).

## RESULTS

The characteristics of the population participating in this study are shown in Table 1. Exposed individuals were classified according to the cleaning labours performed and to their exposure time. Distribution of both genders was equitable in the exposed population and males represented 38% of the control subjects. Age was low in both populations, although the MW group slightly exceeded the mean of 40 years old. Smokers represented 24% of the control group and 42% of the exposed population (range 28–61%). Almost all hired workers wore protective devices (new or reused), but 60% of the volunteers did not use protective clothes and 20% did not use a cellulose mask.

**TABLE 1**
**Characteristics of the Study Population**

| | Controls | Exposed | | | |
|---|---|---|---|---|---|
| | | Total | V | MW | HPW |
| Total number of individuals | 42 | 68 | 25 | 20 | 23 |
| Sex | | | | | |
| Female | 26 | 34 | 15 | 8 | 11 |
| Male | 16 | 34 | 10 | 12 | 12 |
| Age (years)[a] | 22.67 ± 4.50 | 31.99 ± 12.13 | 23.52 ± 7.30 | 41.25 ± 12.16 | 33.18 ± 9.62 |
| Smoking habits | | | | | |
| Nonsmokers | 32 | 39 | 18 | 12 | 9 |
| Smokers | 10 | 29 | 7 | 8 | 14 |
| Use of protective clothes | | | | | |
| New | | 19 | 8 | 8 | 3 |
| Reused | | 33 | 2 | 11 | 20 |
| None | | 16 | 15 | 1 | |
| Use of mask | | | | | |
| New | | 24 | 17 | 3 | 4 |
| Reused | | 35 | 3 | 14 | 18 |
| None | | 9 | 5 | 3 | 1 |

[a] Mean ± SD.

A total number of 66 passive dosimeters were distributed among the different exposure groups to determine the VOC-related characteristics of each zone. Fig. 1 gathers the profiles obtained. They show a major presence of light hydrocarbons as benzene, *n*-heptane, toluene, and *n*-octane in all cases, followed by styrene and *n*-decane, and in the HPW group isopropylbenzene and *n*-propylbenzene. With respect to the comparison of the exposure levels among groups, the highest concentrations took place in the V environment, while the lowest VOC levels were associated with the HPW group. To test if these apparent differences were really significant, an ANOVA test was done comparing TVOC, BTEX, and benzene concentrations as the three representative indices and results are shown in Table 2. These three indices were significantly lower in the hired workers (MW and HPW) than in voluntary individuals, but the first ones did not differ from each other although all values were higher in the MW group.

Regarding to the genetic damage related to the exposure, Fig. 2 and Table 3 show the results of the genotoxicity tests applied. When comparing the total exposed population with controls, just TL distribution showed a displacement to higher values in the exposed. Table 3 reflects this fact as a statistically significant increase of this parameter. Looking at the subgroups of exposure, the MN test only detected a nonsignificant increase in BNMN rate in MW. SCE frequency underwent an increase ($p < 0.01$) associated with the HPW group in relation to controls, and the parameter from the comet assay showed higher levels of DNA strand breaks in V and HPW.

As for the influence of physiologic and consumption parameters (see Tables 4–6), cytogenetic genotoxicity tests have shown higher values for all parameters related to the female gender, but just SCE reached the statistical signification ($p < 0.05$). The comet assay, on the contrary, detected higher DNA damage associated with males in all exposure groups with the exception of MW, where there was no difference between genders. Age has also been shown to influence the damage levels observed. Higher rates of both MN and BNMN were observed in the oldest individuals for all exposed groups and no

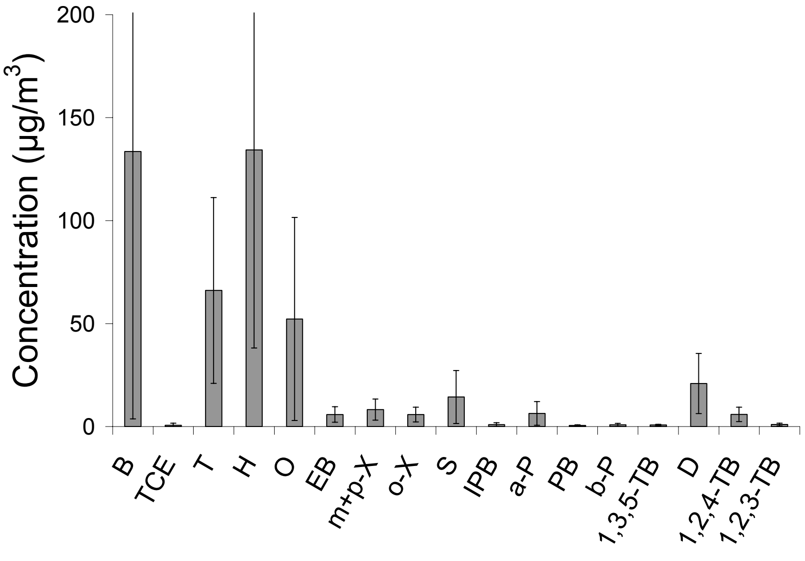

Concentration (µg/m3)
200
150
100
50
0
B
TCE
T
H
O
EB
m+p-X
o-X
S
IPB
a-P
PB
b-P
1,3,5-TB
D
1,2,4-TB
1,2,3-TB

**A**

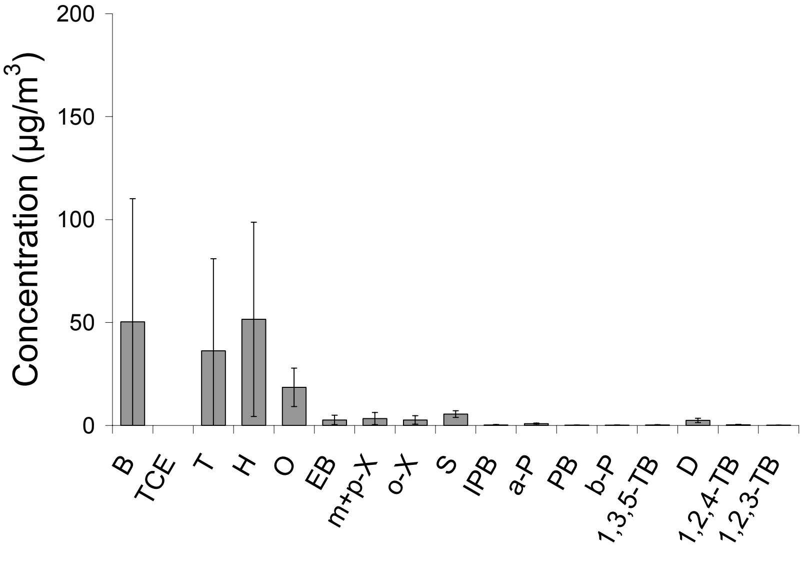

Concentration (µg/m3)
200
150
100
50
0
B
TCE
T
H
O
EB
m+p-X
o-X
S
IPB
a-P
PB
b-P
1,3,5-TB
D
1,2,4-TB
1,2,3-TB

**B**

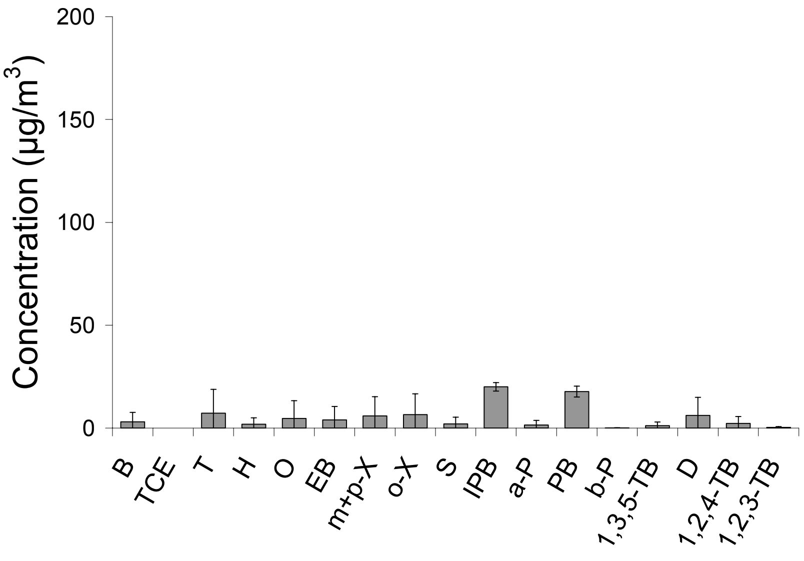

C

**FIGURE 1.** VOC profile obtained from the analyses of dosimeters for each exposed group: (A) V, (B) MW, and (C) HPW. Bars indicate standard deviation. Acronyms used for VOC are shown in Materials and Methods.

differences were obtained in controls. Regarding the SCE results, differences were only detected in the total exposed population and in V, where the frequency of this parameter was increased with age. TL data showed a significant decrease of DNA strand break levels in the oldest group for controls, the total exposed population, and V, while in HPW, increasing age was associated with significantly higher values of TL. Smoking habits seemed not to affect either MN or BNMN rates, but the other cytogenetic biomarker, SCE, showed increases in its frequencies in all exposure groups associated with smokers. Comet assay results are not so uniform, since TL values were significantly increased in smokers belonging to the control and V groups, and no differences were obtained in the total exposed population, MW, and HPW.

Finally, taking into account the possible influence of the protective measures employed by the individuals during the cleaning tasks on the genetic damage (Figs. 3 and 4), it was possible to observe an effect of the use of protective clothes as an increase in SCE frequencies both in V and MW individuals who did not wear these clothes or wore the reused type, and as an increase in TL levels in the same individuals belonging to the MW group. No significant difference was obtained in MN or BNMN frequencies. In the case of the use of a protective cellulose mask, we have only detected a significant increase in SCE frequency in V individuals who did not use it.

**TABLE 2**
**Results Obtained for the Environmental VOC Determination (µg/m³; mean ± SE) in Each Exposure Group**

| | Total VOC | BTEX | Benzene |
|---|---|---|---|
| V | 482.97 ± 62.02 (26) | 196.79 ± 28.77 (25) | 133.57 ± 24.98 (27) |
| MW | 200.85 ± 48.68** (17) | 94.23 ± 28.37* (16) | 50.32 ± 14.51* (17) |
| HPW | 37.81 ± 13.56** (15) | 20.35 ± 7.66** (15) | 3.02 ± 1.18** (15) |

**p* ≤ 0.05, ***p* ≤0 .01, significant difference regarding to the V group. Number is indicated in ( ).

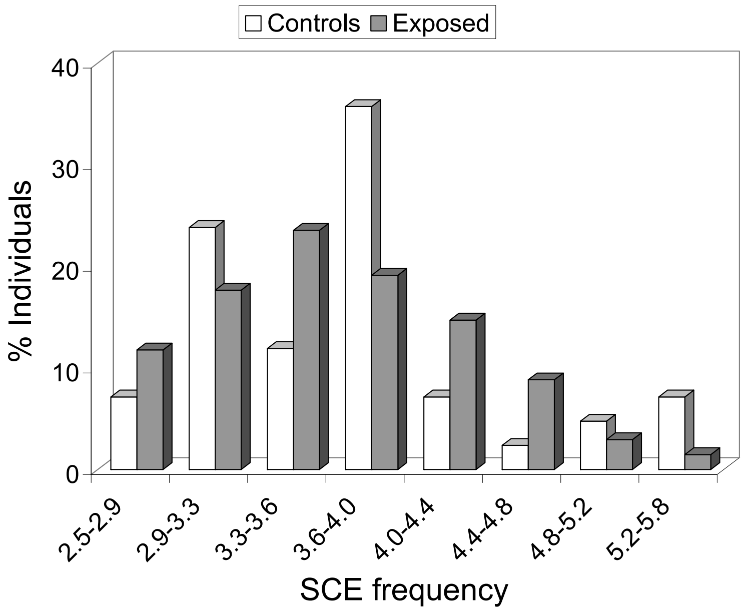

A

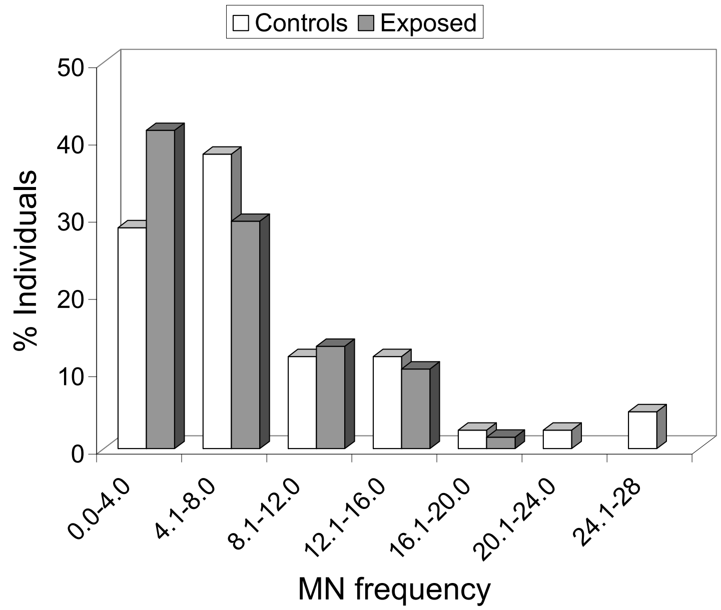

Controls
Exposed
% Individuals
50
40
30
20
10
0
0.0-4.0
4.1-8.0
8.1-12.0
12.1-16.0
16.1-20.0
20.1-24.0
24.1-28
MN frequency

**B**

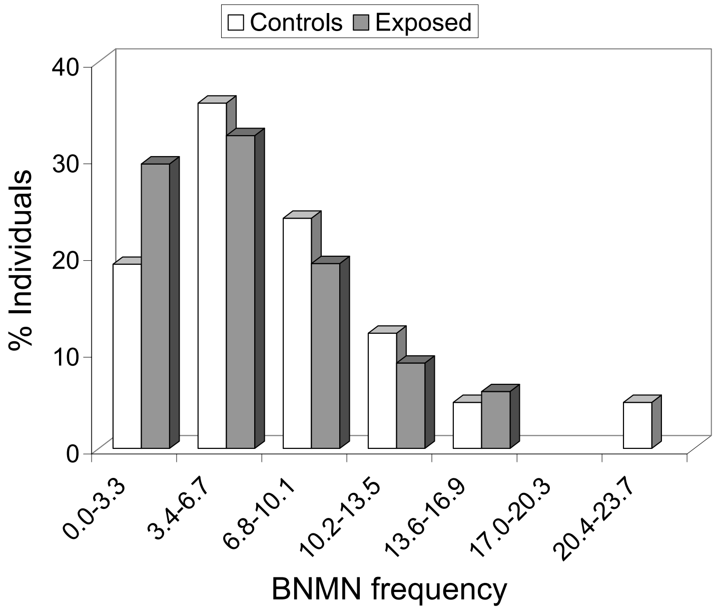

Controls
Exposed
% Individuals
40
30
20
10
0
0.0-3.3
3.4-6.7
6.8-10.1
10.2-13.5
13.6-16.9
17.0-20.3
20.4-23.7
BNMN frequency

**C**

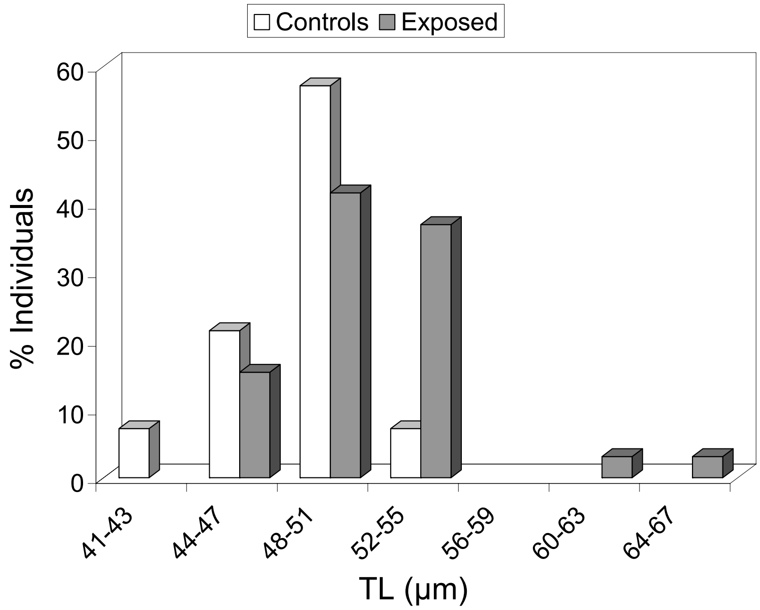

**D**

**FIGURE 2.** Distribution of SCE frequencies (A), MN frequencies (B), BNMN frequencies (C), and TL (D) in the control and exposed populations.

### DISCUSSION

The *Prestige* oil spill was one of the greater environmental disasters of the last decades. A thousand miles of coast were polluted and a large amount of coastal and marine fauna died because of the consequent black tides. Inhabitants of the zone felt helpless as they witnessed not only the damage of their appreciated lands, but also of one of their main sources of financial sustenance. A great number of people came from everywhere to collaborate in the cleaning tasks, together with the workers hired by the autonomic government. From the human health risk point of view, this resulted in a quite large group of people that had undergone different types of exposure, depending on the time spent, the type of task performed, the zone in which they worked, etc. All these characteristics gave rise to the configuration of three exposure groups: volunteers who collaborated in cleaning beaches for 5 days (V), hired individuals who also worked manually on the beaches from the beginning of the spill (MW), and hired subjects that used high-pressure machines to clean the rocks (HPW).

In view of this, the first aim of our work was to characterize in detail the nature and intensity of the exposure that took place in each one of the three different working environments. To this end, we employed 66 passive dosimeters shared among the individuals of the exposed groups. Their analyses brought to light the VOC-concerned characteristics of the air environment. From the qualitative point of view, VOC profiles have shown to be similar among the three groups, the light hydrocarbons being present in a higher proportion in all cases. Besides this, there were quantitatively significant differences according to statistical analyses of the data obtained. In this regard, the higher VOC levels were associated with V environment, followed by MW, and lastly by HPW. In general, concentrations obtained for the two first groups were equivalent to those that could be found in cities with very intense traffic, and higher than those expected in an open environment, such as a beach. VOC values obtained for HPW were lower than for the other two groups, probably due to the use of high-pressure cleaners that may contribute to the dispersion of the compounds or to the fact that the oil deposited on the rocks was more aged. The fact is that actually there is not any normative on the maximum concentrations allowed in this kind of circumstances; nevertheless, taken together, the levels obtained in this study were below the limits

**TABLE 3**
**Effect of Exposure on the Genotoxicity Parameters Evaluated (mean ± SE)**

| | Controls | Exposed (Total) | V | MW | HPW |
|---|---|---|---|---|---|
| SCE | 3.80 ± 0.05 (42) | 3.66 ± 0.03 (68) | 3.41 ± 0.05 (25) | 3.55 ± 0.06 (20) | 4.02 ± 0.06** (23) |
| MN | 8.42 ± 0.53 (42) | 6.23 ± 0.34 (62) | 3.67 ± 0.40 (24) | 8.11 ± 0.71 (18) | 7.60 ± 0.68 (20) |
| CBMN | 7.29 ± 0.41 (42) | 5.79 ± 0.30 (62) | 3.59 ± 0.38 (24) | 7.61 ± 0.65 (18) | 6.85 ± 0.58 (20) |
| TL | 48.79 ± 0.10 (42) | 51.47 ± 0.10** (65) | 53.43 ± 0.19** (23) | 48.17 ± 0.15 (20) | 52.41 ± 0.15** (22) |

***p* < 0.01, significant difference with regard to controls. Number is indicated in ( ).

**TABLE 4**
**Influence of Sex, Age, and Smoking Habits on SCE Frequency (mean ± SE)**

| | Controls | Exposed (Total) | V | MW | HPW |
|---|---|---|---|---|---|
| Sex | | | | | |
| Males | 3.76 ± 0.07 (16) | 3.44 ± 0.04 (34) | 3.13 ± 0.08 (10) | 3.46 ± 0.07 (13) | 3.69 ± 0.08 (11) |
| Females | 3.83 ± 0.06 (26) | 3.87 ± 0.05[a] (34) | 3.59 ± 0.07[a] (15) | 3.71 ± 0.12[b] (7) | 4.33±0.08[a] (12) |
| Age | | | | | |
| <30 years | 3.80 ± 0.05 (40) | 3.58 ± 0.04 (37) | 3.36 ± 0.05 (23) | 3.62 ± 0.13 (5) | 4.05 ± 0.09 (9) |
| ≥30 years | 3.82 ± 0.20 (2) | 3.77 ± 0.05[a] (29) | 3.93 ± 0.18[a] (2) | 3.53 ± 0.07 (13) | 3.99±0.08 (14) |
| Smoking habits | | | | | |
| Nonsmoker | 3.68 ± 0.05 (32) | 3.47 ± 0.04 (37) | 3.36 ± 0.06 (18) | 3.31 ± 0.08 (11) | 3.90 ± 0.09 (8) |
| Smoker | 4.12 ± 0.09[a] (10) | 3.91 ± 0.05[a] (29) | 3.54 ± 0.10 (7) | 3.90 ± 0.10[a] (8) | 4.09 ± 0.07 (14) |

[a]*p* < 0.01, [b]*p* = 0.057, significant difference with regard to reference group (males, <30 years or nonsmokers). *N* is indicated in ( ).

**TABLE 5**
**Influence of Sex, Age, and Smoking Habits on MN and BNMN Frequency (mean ± SE)**

| | Controls | Exposed (Total) | V | MW | HPW |
|---|---|---|---|---|---|
| **MN Frequency** | | | | | |
| Sex | | | | | |
| Males | 8.53 ± 0.85 (17) | 5.72 ± 0.46 (32) | 2.80 ± 0.55 (10) | 7.45 ± 0.88 (11) | 6.64 ± 0.86 (11) |
| Females | 8.40 ± 0.68 (25) | 6.77 ± 0.51 (30) | 4.29 ± 0.57[d] (14) | 9.14 ± 1.21 (7) | 8.78±1.08 (9) |
| Age | | | | | |
| <30 years | 8.60 ± 0.55 (40) | 3.97 ± 0.34 (35) | 3.39 ± 0.40 (23) | 4.75 ± 1.09 (4) | 5.25 ± 0.81 (8) |
| ≥30 years | 5.50 ± 1.65 (2) | 9.04 ± 0.64[a] (26) | 10.00 ± 3.15[a] (1) | 9.07 ± 0.86[b] (14) | 8.91±1.02[a] (11) |
| Smoking habits | | | | | |
| Nonsmoker | 9.30 ± 0.66 (30) | 6.12 ± 0.46 (33) | 3.88 ± 0.51 (16) | 8.10 ± 0.95 (10) | 8.43 ± 1.23 (7) |
| Smoker | 6.33 ± 0.84 (12) | 6.34 ± 0.50 (29) | 3.25 ± 0.66 (8) | 8.13 ± 1.08 (8) | 7.15 ± 0.81 (13) |
| **BNMN Frequency** | | | | | |
| Sex | | | | | |
| Males | 7.18 ± 0.65 (17) | 5.25 ± 0.40 (32) | 2.70 ± 0.52 (10) | 6.91 ± 0.79 (11) | 5.91 ± 0.73 (11) |
| Females | 7.36 ± 0.54 (25) | 6.37 ± 0.46[c] (30) | 4.14 ± 0.54[e] (14) | 8.71 ± 1.11 (7) | 8.00±0.94[f] (9) |
| Age | | | | | |
| <30 years | 7.38 ± 0.43 (40) | 3.89 ± 0.33 (35) | 3.26 ± 0.38 (23) | 4.75 ± 1.09 (4) | 5.25 ± 0.81 (8) |
| ≥30 years | 5.50 ± 1.65 (2) | 8.23 ± 0.56[a] (26) | 10.00 ± 3.15[a] (1) | 8.43 ± 0.77[b] (14) | 7.82±0.84[b] (11) |
| Smoking habits | | | | | |
| Nonsmoker | 8.00 ± 0.51 (30) | 5.70 ± 0.41 (33) | 3.75 ± 0.48(16) | 7.60 ± 0.87 (10) | 7.43 ± 1.03 (7) |
| Smoker | 5.50 ± 0.68 (12) | 5.90 ± 0.45 (29) | 3.13 ± 0.62(8) | 7.63 ± 0.97 (8) | 6.54 ± 0.71 (13) |

[a]*p* <0 .01, [b]*p* < 0.05, [c]*p* = 0.067, [d]*p* = 0.069, [e]*p* = 0.064, [f]*p* = 0.075, significant difference with regard to reference group (males, <30 years or nonsmokers). Number is indicated in ( ).

**TABLE 6**
**Influence of Sex, Age, and Smoking Habits on DNA Damage (TL [µm], mean ± SE)**

| | Controls | Exposed (Total) | V | MW | HPW |
|---|---|---|---|---|---|
| Sex | | | | | |
| Males | 49.76 ± 0.20 (16) | 52.63 ± 0.18 (33) | 56.71 ± 0.08 (9) | 48.96 ± 0.23 (12) | 53.23 ± 0.26 (12) |
| Females | 49.13 ± 0.15[a] (26) | 52.01 ± 0.17[b] (32) | 52.94 ± 0.07[a] (14) | 49.16 ± 0.12 (8) | 53.00±0.08[a] (10) |
| Age | | | | | |
| <30 years | 49.48 ± 0.12 (40) | 53.09 ± 0.16 (38) | 54.58 ± 0.23 (22) | 48.91 ± 0.27 (6) | 52.33 ± 0.28 (10) |
| ≥30 years | 47.29 ± 0.66[a] (2) | 51.24 ± 0.18[a] (27) | 50.66 ± 0.69[a] (1) | 49.10 ± 0.24 (14) | 53.78 ± 0.26[a] (12) |
| Smoking habits | | | | | |
| Nonsmoker | 48.82 ± 0.14 (31) | 52.49 ± 0.16 (39) | 53.82 ± 0.24 (18) | 50.00 ± 0.27 (12) | 53.11 ± 0.32 (9) |
| Smoker | 50.92 ± 0.22[a] (11) | 52.08 ± 0.19 (26) | 56.51 ± 0.57[a] (5) | 47.61 ± 0.23 (8) | 53.13 ± 0.23 (13) |

[a]*p* < 0.01, [b]*p* <0 .05, significant difference with regard to reference group (males, <30 years or nonsmokers). Number is indicated in ( ).

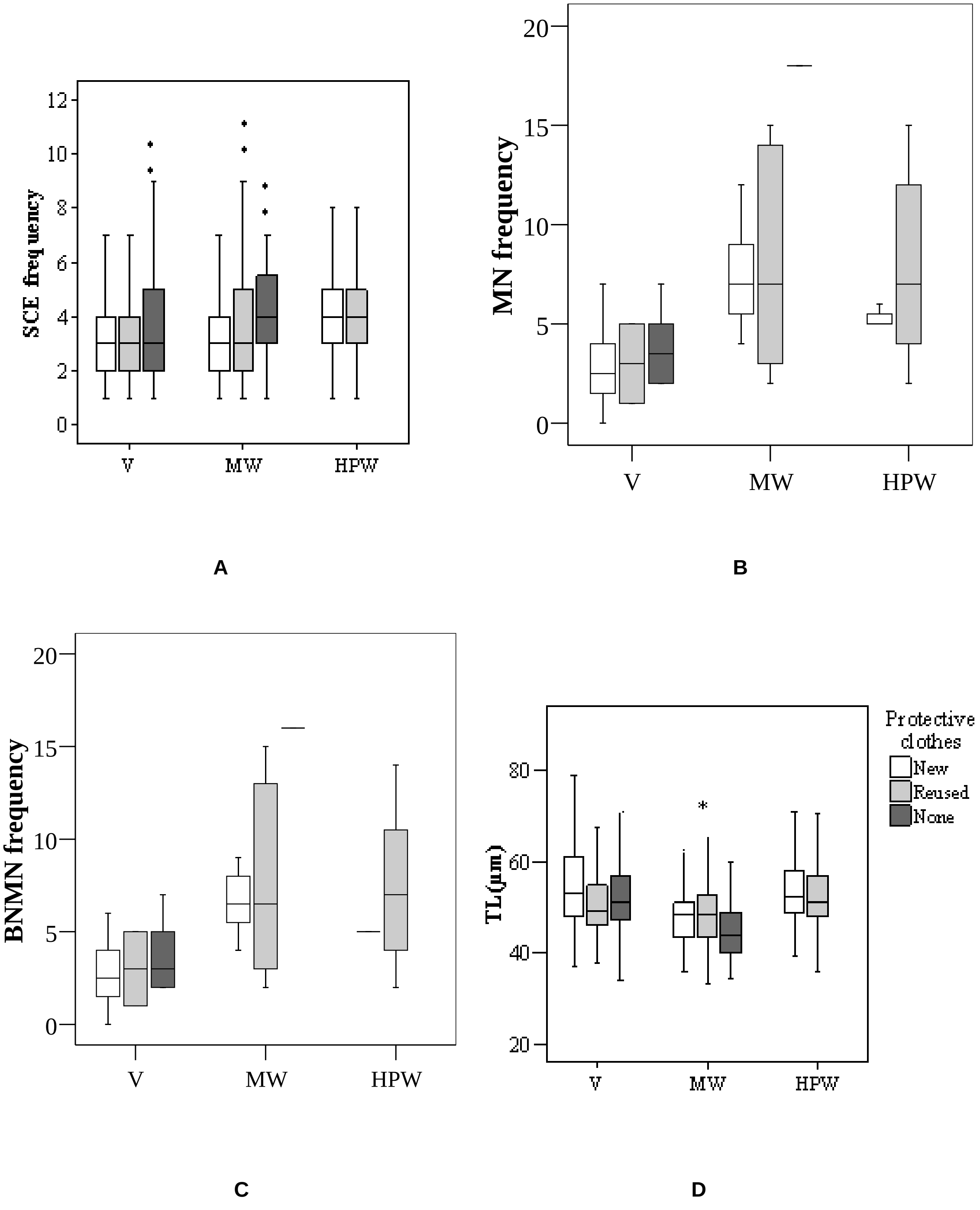

**FIGURE 3.** Boxplots showing SCE frequencies (a), MN frequencies (b), BNMN frequencies (c), and TL (d) in the exposed groups according to the use of protective clothes. *$p < 0.05$, **$p < 0.01$, significant difference with regard to the use of new devices.

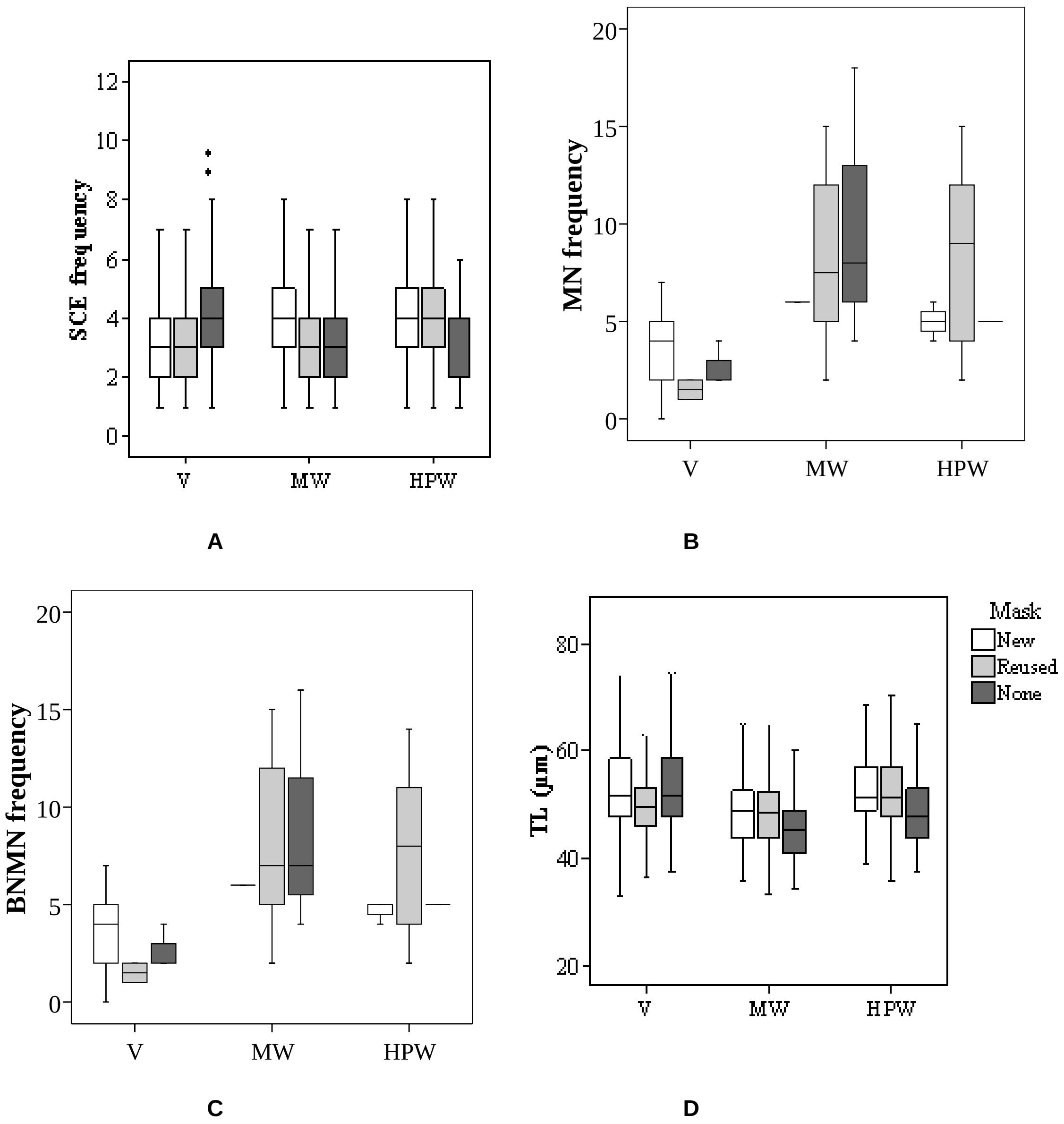

**FIGURE 4.** Boxplots showing SCE frequencies (a), MN frequencies (b), BNMN frequencies (c), and TL (d) in the exposed groups according to the use of protective mask. $^{**}p < 0.01$, significant difference with regard to the use of new devices.

established by the Spanish *Instituto Nacional de Seguridad e Higiene en el Trabajo*[11] and by the American Conference of Governmental Industrial Hygienists[12] in reference to closed environments.

With regard to the genotoxicity biomarkers, it seems that the comet assay was the most sensitive test to detect the effect of the exposure to the *Prestige* oil, showing increases in DNA strand break levels in the groups of V, HPW, and in the total exposed population. The cytogenetic tests, on the other hand, did not detect increases in the frequency parameters related to the exposure, with thr exception of the SCE rate in the HPW group that underwent an increase compared to controls. MN and BNMN did not experience variations with the exposure, only a slight increase of the second variable was observed in MW, but it did not reach statistical significance. These results agree with our previous finds in a population of *Prestige* oil-exposed volunteers engaged in the autopsies and cleaning of oil-contaminated birds[13], and might indicate that the damage induced in the individuals was a short-term damage that could be easily repairable and therefore it was not finally fixed. To the best of our knowledge, this is the first study on the genotoxic damage caused in humans by oil exposure after an accidental spill. However, there are several reports dealing with different PAH exposures that also observed increases in comet assay parameters[14,15].

When evaluating an exposure event and its repercussions on DNA integrity, there are several factors that must be taken into account since they may affect the results obtained in any way. Among them sex, age, and tobacco consumption are the most studied, the last one being especially related to oil exposure because of the parallelism existing in their composition.

Almost all genotoxicity epidemiological studies include sex/gender among their influencing factors, the conclusions being diverse. More converging results revolve around cytogenetic tests, accepting a general increase of damage in females. Lazutka et al.[2] established this difference in a rate of 7.5% for SCE assay. Our results from the cytogenetic tests agreed with this tendency, especially in SCE test where statistical signification was reached in almost all groups. Just the opposite happened with comet assay data, since the differences were shown as increases in TL related to males belonging to the total exposed population, V, and HPW. Contradictory results are reported[16,17,18] and the interaction of all the influencing factors that may act as confounding elements should be noticed when analyzing the data giving rise to the different results obtained.

It is well known that DNA experiences an error and damage accumulation process with time, mainly due to loses in the efficiency of the repair mechanisms. Our results reflected this effect showing increases in SCE, MN, and BNMN frequencies associated with the oldest exposed individuals and agreeing with several previous reports[19,20,21]. This phenomenon did not happen in controls, probably indicating a potential susceptibility component, older individuals being more prone to be genetically damaged by the *Prestige* oil. Nevertheless, results from the comet assay are only concordant with this in the case of HPW, following data from controls, V, and the total exposed population the opposite tendency. Perhaps, the most plausible explanation for this controversy resides in the fact that part of the strand breaks detected by this technique comes from incomplete excision repair phenomena, and they are probably more active in younger individuals. On the other hand, Kim et al.[22] and Sul et al.[23] reported no influence of this factor on comet assay results.

Tobacco consumption is assumed as a risk factor in mutagenesis and carcinogenesis processes due to the fact that there are more than 40 carcinogenic substances in its composition[24]. Looking at the results, the MN test did not reflect any influence of this factor in any of its two parameters, agreeing with data reported by Barale et al.[19] and Duffaud et al.[25]. On the contrary, a smoking effect has been detected in SCE and comet assay as increases in SCE frequency and TL in smoker individuals with regard to nonsmokers in almost all groups of individuals. These results are concordant with those reported by some other studies dealing also with these biomarkers[26,27,28].

Finally, attending to the cleaning conditions, we have taken into consideration the protective measures employed while developing the tasks. The use of waterproof overalls seemed to exert a certain protective effect, detected in SCE and comet assay, but the use of cellulose masks scarcely modified the results of the genotoxicity tests applied. This leads us to think that maybe the kind of protective devices employed by the individuals were not the most adequate, or that there were other factors with more weight on the genetic response.

In conclusion, in this work we have evaluated the extent of the exposure that took place in the different environments of the polluted zones and estimated the magnitude of the toxic effect induced by the exposure at the genetic level. We have observed a considerable presence of VOC in the environmental air, especially for the V group. Oil exposure during the cleaning tasks caused an increase in the genotoxic damage in individuals, the comet assay being the most sensitive biomarker to detect it. Sex, age, and tobacco consumption were shown to influence the level of genetic damage, while the effect of using protective devices was smoother than expected. Taking all this into account, it can be stated that these kinds of studies are necessary not only for evaluating the health risks associated with the exposure, but also for defining all the variables that must be considered when designing a similar study.

### ACKNOWLEDGEMENTS

This work was funded by a grant from the Xunta de Galicia (PGIDT04PXIB10602PR), a grant from the Instituto Nacional de Seguridad e Higiene en el Trabajo (04-12752-UCO-4), and from the Fundación Arao. B. Pérez-Cadahía has been supported by a fellowship from the University of A Coruña.
